# Predictive Role of p53 Protein as a Single Marker or Associated to Ki67 Antigen in Oral Carcinogenesis 

**DOI:** 10.2174/1874210600802010024

**Published:** 2008-02-21

**Authors:** L. Montebugnoli, L. Felicetti, D.B. Gissi, F. Cervellati, D. Servidio, C. Marchetti, C. Prati, F. Flamminio, M.P. Foschini

**Affiliations:** 1 Department of Oral Science, University of Bologna; 2 Section of Anatomic Pathology at Bellaria Hospital, University of Bologna, Italy

## Abstract

p53 over-expression has been proposed as a reliable marker associated to oral carcinogenesis, although only about 50% of oral carcinomas (OSCC) are associated with p53 over-expression and even p53-negative lesions can progress to OSCC. The aim of the study was to determine whether the combination of p53 over-expression and p53 low-expression associated with Ki67 over-expression (high Ki67/p53 ratio) could lead to a more sensitive parameter. Immunohistochemical expression of Ki67 and p53 was measured in 54 specimens from OSCC; 27 specimens from moderate/severe epithelial dysplasia; 32 specimens from oral leukoplakias without epithelial dysplasia, and 13 specimens with normal epithelium. p53 over-expression was found in 31 (53%) samples from OSCC, in 10 (37%) samples from severe dysplasias, and in 5 (15%) samples from non-dysplastic lesions, while the combination of high p53 values with high Ki67/p53 ratio was observed in 93% of OSCC, in 81% of dysplastic lesions, and in 50% of non-dysplastic lesions. This parameter may have a clinical implication to detect early lesions with an impairment of p53 pathway, and probably at risk of progress to OSCC.

## INTRODUCTION 

It has been widely demonstrated that most oral squamous cell carcinomas (OSCC) develop through their precancerous steps which are defined as potentially malignant lesions and are histologically characterized by increasing grades of epithelial dysplasia [1-4].

So far, the only accepted method to quantify the risk of progression to OSCC of a potentially malignant lesion, is the presence/absence of histological findings of dysplasia [5-6], although the presence of dysplasia does not always indicate malignant transformation and its absence does not preclude it [7]. Moreover, histological assessment of dysplasia is extremely subjective and prone to inter and intra-observer variation [8], and additionally some potentially malignant lesions do not show dysplastic alterations [9-10].

These considerations have stimulated intense research in this area, and many studies have been conducted so far, in an attempt to find out molecular markers that are associated with OSCC and that can predict malignant transformation when found in epithelial precursor lesions, especially in cases without signs of dysplasia [11-13].

The immunohistochemical analysis of p53 protein is a simple and inexpensive method that has been widely used for this purpose, and many studies have shown that it is involved in oral carcinogenesis and its alteration occurs early in the process of neoplastic transformation, often preceding recognizable histological alterations [12-14-16].

p53 over-expression has been widely demonstrated to be a reliable predictor of progression to OSCC, either if we consider the over-expression as a physiologic response to induce cell cycle arrest in genetically altered hyper-proliferating cells or as a primary genetic defect leading to the production of high levels of mutated non-functional p53 protein; this is why the most utilized antibodies in the literature stain the total form of p53 protein and can not discriminate between wild or mutated form [17-18].

However, p53 over-expression as a marker of progression to OSCC has not been considered highly sensitive, probably because only about 50% of OSCC are associated with over-expression of p53 protein [19-22].

It follows that if the occurrence of p53 over-expression can be considered specific in detecting oral lesions at risk of developing p53-positive tumours, the presence of low p53 values should be considered as non-informative for prognostic purposes, since it can not distinguish between lesions with a genetically reduced production of p53 at high risk of developing p53-negative tumours, and lesions with a physiological low p53 activity or lesions that follow different carcinogenic pathways in which the p53 abrogation does not play a role [7,11-22].

It is well known that in non-neoplastic cells the expression of p53 is usually related to the cell proliferating rate, since p53 is considered as having an “oncogene checkpoint” function that guards cells against hyperproliferative signals [23].

It follows that in non-neoplastic cells that respond to proliferative signals from inflammatory changes, traumas or hypoxia, a proportional increase in p53 activity should be expected [24-25].

Thus, we may assume that the finding of an increased proliferating rate associated to a depressed p53 expression (increased mitotic/apoptosis ratio) in potentially malignant lesions might possibly identify genetically damaged lesions lacking of p53 protective role during cell cycling and may be at risk of progressing to p53-negative cancer.

In the present study, the Ki67 protein has been utilized as a immunohistochemical marker of proliferating cells [26], and samples with high Ki67/p53 ratio scores were taken into consideration together with samples with high p53 scores to determine whether this combination of scores could be useful to discriminate oral lesions with an impaired cell turnover. 

## MATERIALS AND METHODOLOGY

A total of 126 biopsy samples from 126 consecutive patients who have referred to the Department of Oral Sciences of the University of Bologna, Bologna (Italy) between January 2004 to September 2006 were analyzed: 54 specimens from infiltrating OSCC, 27 from moderate/severe epithelial dysplasias (19 from non-tumoral areas adjacent to OSCC included in the study, and 8 from oral leukoplakias), 32 from oral leukoplakias with epithelial hyperplasias and no signs of dysplasia, and 13 from normal epithelium oral mucosa obtained during third molar removal or as part of the excision of benign conditions. All samples underwent histological and immunohistochemical analysis.

Histological diagnoses were performed at the Department of Pathology of the University of Bologna, at Bellaria Hospital (Bologna, Italy), following the criteria describe in the WHO book. Specifically moderate\severe dysplasia (atypical hyperplasia according to the Ljubljiana classification) was characterized by increasing atypia, loss of polarity, and frequent mitoses, involving more than two-third of the epithelium while lacking infiltrative growth. Squamous cell Hyperplasia (Simple hyperplasia, according to the Ljubljiana classification) was characterized by increased basal-parabasal layers, acanthosis, in absence of architectural alterations [6]. No cases of mild dysplasia were included in the study. The rationale to only select lesions with high grades of dysplasia, was to be sure about the effective presence of dysplasia by reducing the subjectivity of its assessment and their well established relative high rate of malignant transformation [8,27]; this to well differentiate the group of lesions with dysplasia from that of hyperplastic lesions.

Immunostaining was performed on 2-µm thick sections serially cut from the selected blocks. The following antibodies were employed: monoclonal anti-Ki67 (Dako, Denmark, clone MIB-1, diluted 1:200) and monoclonal anti-p53 (Dako, clone p53, diluted 1:50). The processing was performed in an automatic stainer (Autostainer, Ventana, USA). All the cases contained an internal control as basal cells of the oral epithelium show nuclear positivity for the two markers under study. Negative controls consisted in omitting the primary antibody. Counting the percentage of positive nuclei in 400 consecutive epithelial cells of selected areas representative of the lesion gave a semi-quantitative evaluation of the immunohistochemical results. Fig. (**1**) shows an example of positive and negative staining of p53 protein.

### Statistical Analysis

One-variable analysis was performed on p53 values and Ki67/p53 ratio to collect summary statistics, and density trace diagrams were used to obtain the shape of distribution of the data.

We established the cut-off value for p53 and Ki67 over-expression as 20% staining and for high Ki67/p53 ratio as 3 because no sample from normal mucosa showed higher values both in the present and in a previously published study [24].

Thus high p53 (p53 over-expression) were considered when equal or more than 20%, while high Ki67/p53 ratio was considered when equal or more than 3.

A multiple logistic regression model was fitted to describe the relationship between the presence/absence of dysplasia (dependent variables) or between dysplasia and OSCC (dependent variables)and 2 independent variables: p53 over-expression versus p53 low expression, and the combination of samples with high p53 and samples with high Ki67/p53 ratio versus low p53 and low Ki67/p53 ratio.

One-way ANOVA was used to compare p53 mean values (continuous dependent variable) between samples from OSCC, epithelial hyperplasia, epithelial dysplasia and controls (nominal independent variable). Fisher’s least significant difference procedure (LSD) was the method to discriminate among the means at 95.0% confidence level.

## RESULTS

**Fig. (2).**
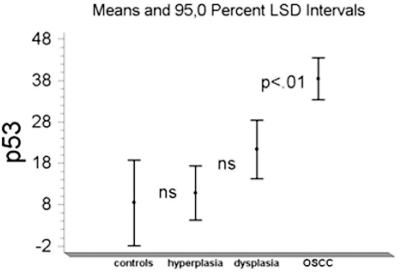
Mean p53 values in controls, hyperplasia, dysplasia and OSCC.

One-way ANOVA showed that the mean p53 value in OSCC (38.4±34.9) was significantly higher (F=8.8; p<.01) than those found in controls and in oral lesions either with or without dysplasia (respectively, 8.3± 3.5, 21.3±27.1, and 10.8 ±6.9). Fisher’s least significant difference procedure did not show any difference between the last three groups (Fig. **2**). The wide Standard Deviation in samples from OSCC and oral lesions with dysplasia is the consequence of the contemporary presence of high and low p53 values in these oral lesions as documented by the density trace diagrams in Fig. (**3**).

**Fig. (3).**
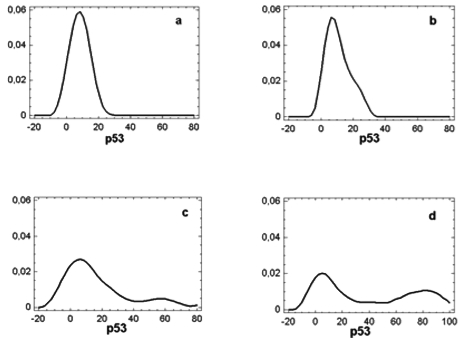
Density trace diagrams showing p53 distribution in specimens from normal mucosa **(a)**, epithelial hyperplasias **(b)**, epithelial dysplasias **(c)** and OSCC **(d)**.

Normal mucosa: the density trace diagram from normal samples showed a shape of distribution of p53 values characterized by a single peak with a median of 10% (range 5-15), and values always less or equal to 15%. The sample come from a normal distribution with a standardized skewness (.74) and kurtosis (-.17) values within a range expected from a normal distribution (Fig. **3a**).

The Ki67/p53 ratio showed a range from 0.5 to 1.5 with a median of 1 and values never exceeding 2.5.

OSCC: the density trace diagram from OSCC showed a shape of distribution of p53 values characterized by two well defined peaks that distinguished two very different population where high or low p53 values were expressed respectively in 31 (57%) and 23 (43%) samples (Fig. **3d**).

A Ki67/p53 ratio equal or more than 3 was found in 19 patients out of 23 (83%) showing low p53 values (Table **1**). All samples with high p53 values had low Ki67/p53 ratios.

Epithelial Dysplasia: similarly to OSCC the shape of distribution of p53 values showed two well defined peaks where high values are expressed respectively in 10 (37%) samples of which 8 (42%) from non-tumoral areas adjacent to OSCC (Fig. **3c**). High Ki67/p53 ratio was found in 12 out of 17 (71%) samples with low p53 values of which 9 (82%) from margins of OSCC (Table **1**). All samples with high p53 values had low Ki67/p53 ratios.

Epithelial Hyperplasia: high p53 values were found in 5 (15%) samples (Fig. **3b**), and high Ki67/p53 ratio was found in 11 out of 27 (37%) samples with low p53 values (Table **1**). Again, all samples with high p53 values had low Ki67/p53 ratios.

The combination of high p53 values with high Ki67/p53 ratio was observed in 93% (50 out of 54) of OSCC and in 81% (22 out of 27) of epithelial dysplasias, of which 89% (17 out of 19) from non-tumoral areas adjacent to OSCC, and in 50% (16 out of 32) of epithelial hyperplasias, while it was never observed in any sample from normal mucosa. Table **1** summarizes the results.

The results of fitting a multiple logistic regression model to describe the relationship between the presence/absence of dysplasia and the 2 independent variables (p53 over-expression versus p53 low expression, and the combination of samples with high p53 and samples with high Ki67/p53 ratio versus low p53 and low Ki67/p53 ratio) showed that the only parameter related to the presence/absence of dysplasia and reaching the significance (Chi square 6.6; p<.01) was the presence/absence of samples with high p53 associated with samples with high Ki67/p53 ratio.

No significant difference was found between OSCC and epithelial dysplasia when this combination was utilized (Chi square .31; ns).

## DISCUSSION

It is widely accepted that OSCC develops as a result of accumulation of genetic errors in the same tissue [28,29], and that mutations in the TP53 gene leading to loss of function is one the most common genetic damage found in human tumour and in OSCC [30-32].

TP53 mutations may result in a over-production of p53 inactive proteins which accumulate in the epithelium either due to blocking by another protein or due to partial degradation [32], or instead in a abrogation of p53 wild function by epigenetic mechanisms, due to a mutation that did not result in a stable form of the p53 protein [33]. This can be well evidentiated by immunohistochemical analysis of paraffin embedded specimens showing staining of a high number of neoplastic cells in the first event or complete negative staining in the latter cases [34].

The shape of distribution of p53 values from OSCC in the present study, is in line with this statement, clearly showing the presence of two well defined peaks that distinguish two very different OSCC population, where high or low p53 values were expressed respectively in about 57% and 43% of cases. The prevalence of higher p53 values in the entire population explains the statistical difference in the mean p53 values between malignant samples and samples from normal mucosa.

The evidence that the p53 pathway is very important in the biology of oral cancer has led to the application of such immunohistochemical analysis as a simple rapid and inexpensive method to potentially malignant lesions in an attempt to find a useful marker to predict progression to OSCC.

Higher mean p53 values with respect to normal mucosa has been demonstrated in lesions with and without signs of dysplasia and it has been documented that lesions progressed to OSCC showed p53 over-expression early during the process, although most of the studies in the literature have utilized antibodies that can not discriminate between wild or mutated form [13,35-36]. Nevertheless, studies that calculate the positive and negative predictive value are still lacking.

In our study we did not find significantly higher p53 mean values in potentially malignant lesions with respect to normal mucosa although samples from lesions with severe dysplastic alterations showed higher mean p53 value than normal mucosa, but did not reach statistical significance. The limited number of controls may have contributed to hide a significant difference between p53 mean values from potentially malignant lesions and normal mucosa, although all p53 values from normal mucosa kept lower than the cut-off considered in the present and in a previously published study [24].

However, p53 analysis did reveal differences between our potentially malignant lesions and normal mucosa since about 40% of sample in the group of dysplastic lesions and 15% in the group of leukoplakias without dysplastic alterations showed p53 values higher than the maximum value found in normal mucosa. We suppose that the prevalence of normal or low values in dysplastic lesions can explain the lack of statistical difference in the mean p53 values between pre-malignant lesions and normal mucosa.

When concerning lesions with low p53 expression, the present data showed that low staining was present in more than 40% of OSCC, and even in about 60% of lesions with severe dysplasia, most of them at margins of OSCC and probably precursors of p53-negative tumours [7].

If we take the p53 over-expression as a predictive marker, the occurrence of positive samples in our population is probably too low to discriminate lesions at “high risk” of malignancy, also considering that not all p53-positive lesions will progress to cancer [37].

Furthermore, if we consider samples with low p53 expression as lesions “not at risk” of developing cancer, the occurrence of p53-negative lesions in our population of severe epithelial dysplasias is too high, considering that most of them were situated at margins of OSCC and thus at very high risk of malignant changes [38-39].

These data underline the low predictive value of p53 when used as a single marker, since the high occurrence of low p53 staining in pre-malignant lesions is very difficult to be interpreted and not enough to rule out the progression to OSCC.

This is in agreement with many reports that have stated that p53 has a good specificity, i.e the likelihood of malignant transformation is high when it is over-expressed, but a low sensitivity since the absence of p53 staining did not preclude malignant transformation in a considerable proportion of pre-malignant lesions [20,40-41].

Instead, in the presence of a lesion in predicate to develop cancer, the avoidance of false-negative results takes priority, so as not to miss patients who actually have a disease at risk of developing OSCC; in this situation, the sensitivity is most important to maximize negative-predictive accuracy, i.e. the likelihood that a negative test relates to the absence of any risk of malignancy [42].

To this direction have gone our results obtained by the combination of samples with high p53 values and samples with low p53 values but high Ki67 values, i.e. a high Ki67/p53 ratio.

We utilized this approach to see whether the presence of an increased mitotic activity not associated to an increased p53 activity might discriminate lesions with high proliferative activity where the presence of a genetic damage prevents a reactive stimulation of p53.

The rationale was that in non-neoplastic cells, an increased cell proliferation due to a series of factors such inflammation, trauma etc, usually elicits a p53 protein accumulation that is considered to be a physiologic response to the increased proliferation rate of keratinocytes as a guardian of epithelial growth [43-44].

In the present study, high Ki67/p53 ratios were found in 83% of OSCC with low p53 expression and even in 71% of severe epithelial dysplasias with low p53 values, and the combination of samples with p53 over-expression and samples with low p53 values but high Ki67/p53 ratio, led to a very high association with OSCC and epithelial dysplasias. About 90% of both OSCC and epithelial dysplasias were positive to this combination, while normal epithelium never was.

Dysplasia is still now the most important predictive factor providing a surgical or non-surgical therapeutic approach. However histological assessment of dysplasia is extremely subjective [8], and some potentially malignant lesions do not show dysplastic alterations [9-10]. For these reasons, it would be very important to found alternative parameters that allow an early detection of pre-neoplastic lesions without any dysplastic changes. The evaluation of p53 associated with Ki67, when applied to potentially malignant lesions without features of dysplasia could be useful to enable the detection of cell alterations in such lesions.

In the present study, the combination of p53-over-expression and high Ki67/p53 ratio was present in about 50% of leukoplakias without signs of dysplasia.

This percentage surely overestimates the risk of developing cancer, but could be useful to increase the predictive sensitivity and to rule out at least 50% of lesions with a presumably very low risk of developing OSCC.

## CONCLUSIONS

p53 immunostaining as a single marker can not be considered a sensitive marker that would help to assess the degree of cell alteration in oral lesions at risk of developing cancer.

While it could be accepted that p53 over-expression in potentially malignant lesions may indicate a risk of developing cancer, the occurrence of low p53 staining is very difficult to be interpreted and does not rule out a possible link to oral carcinogenesis.

In the present study the combination of p53 over-expression and high Ki67/p53 ratio seemed to better associate with the occurrence of OSCC and severe epithelial dysplasia, and may have a routine implication as an easy method to early detect oral lesions with an impairment of p53 pathway, and probably at risk of developing OSCC.

## Figures and Tables

**Fig. (1).**
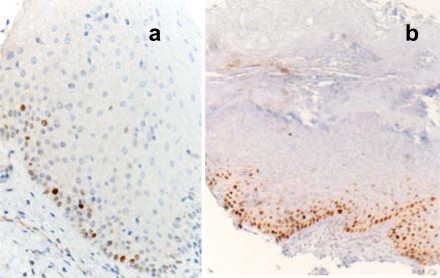
In normal oral mucosa p53 positivity is limited to rare basal keratinocytes **(a)**; in this case with marked hyperkeratosis the p53 antibody stains the nuclei of numerous basal and suprabasal keratinocyte **(b)**.

**Table 1.** Number (%) of Specimens with p53 Over-Expression and Specimens with p53 Over-Expression Associated with Samples with High Ki67/p53 Ratio from Normal Mucosa, Epithelial Hyperplasias, Epithelial Dysplasias and OSCC

	Normal Mucosa	Epithelial Hyperplasias	Epithelial Dysplasias	OSCC
number	13	32	27	54
Samples with p53 over-expression	0 (0%)	5 (15%)	10(37%)	31(57%)
Samples with p53 over-expression associated with samples with high Ki67/p53 ratio.	0 (0%)	16 (50%)	22 (81%)	50(93%)
